# Young TPI: empowering animal-free science among the next- generation of scientists

**DOI:** 10.3389/ftox.2024.1521317

**Published:** 2025-01-15

**Authors:** Marta G. Valverde, Fatima Zohra Abarkan, Rebecca Van Eijden, Julia M. L. Menon, Nikolas Gaio, Aarti Ramchandran, Victoria C. De Leeuw

**Affiliations:** ^1^ Founding Board Member of Young TPI, The Hague, Netherlands; ^2^ Division Pharmacology, Utrecht Institute for Pharmaceutical Sciences, Utrecht, Netherlands; ^3^ Faculty of Science, Radboud University, Radboud Honours Academy, Nijmegen, Netherlands; ^4^ Institute for Management Research, Radboud University, Radboud Honours Academy, Nijmegen, Netherlands; ^5^ Preclinicaltrials.eu, Netherlands Heart Institute, Utrecht, Netherlands; ^6^ BIOND Solutions B.V., Delft, Netherlands; ^7^ BioTechnology Solutions, MSD Animal Health Netherlands, Boxmeer, Netherlands; ^8^ Centre for Health Protection, National Institute for Public Health and the Environment (RIVM), Bilthoven, Netherlands

**Keywords:** young, network, animal-free scientific research, transition, innovation, empowering, visibility, collaboration

## Abstract

Strategies emphasizing animal-free innovation are imperative for the contemporary and future scientific research. They not only address important ethical concerns, but also should directly improve research accuracy and reliability through redirecting scientific inquiry toward more reliable and translatable methodologies. Promotion and encouragement for use of animal-free innovations among the next-generation of scientists, alongside knowledge acquisition and training in the increased capabilities of novel technologies, are fundamental for advancing science and the welfare of animals used for scientific purposes. The Dutch government has promoted initiatives such as *Transitie Proefdiervrije Innovatie* (TPI) to make the public aware of the current situation. However, the transition towards animal-free innovations will span over more than two generations. In this context, Young TPI emerged as the-first-of-its-kind network comprising young professionals and students dedicated to revolutionizing scientific practices by catalyzing the shift towards animal-free research. Grounded on three pillars - collaboration, awareness-raising, and networking - Young TPI has evolved into a premier youth network in the Netherlands. Boasting over 270 members spanning Dutch 49 institutions, including biotechnology startups and pharmaceutical companies and universities, Young TPI harnesses the diverse expertise of its members to propel a sustainable, future-proof transition and to promote a continuous dialogue with a wide range of stakeholders. This manuscript describes the conception, establishment, and progress of Young TPI from its start to present, detailing its strategy for communication, activities, and funding mechanisms, and ongoing endeavors to enlist new members and forge strategic alliances in pursuit of its mission.

## 1 Introduction

The conversation on animal research ethics traces back to 1959, when the triad of terms “Replacement, Reduction and Refinement” (“the 3R’s” or “Three R’s”) was introduced, igniting the discussion on considering the welfare of animals used in research and advocating for humane treatment (Russell and Burch, 1959). Over the years, the 3R’s have been globally adopted and recognized by scientific communities, shaping national and international laws in favor of animal welfare (Broom, 2017).

More recently, the longstanding practice of utilizing animals in scientific research is facing increased scrutiny, with debates centered around the applicability and translatability of animal models to human conditions. The surge in ethical concerns and an enhanced understanding of animal welfare, together with a plethora of models with better predictive capabilities, have prompted initiatives toward animal (testing)-free innovations (Rand, 2022; Balls et al., 2024).

Non-animal methods (NonAMs) encompass a diverse spectrum of technologies, methodologies, approaches, and their combinations, offering insights into chemical hazard and risk assessment for human safety without relying on animal use. This umbrella includes *in silico* and *in vitro* techniques (Stucki et al., 2022). The novelty in NonAMs extends beyond their development; it lies in their innovative application within a regulatory framework and as replacements of conventional testing prerequisites (Grimm et al., 2023; Stresser et al., 2023). However, despite the enthusiasm for proposing NonAMs, the field encounters two significant barriers. Firstly, there is a predominant focus on developing new methods, which leads to a lack of validation and implementation of existing NonAMs (Abarkan et al., 2022). Secondly, validation and implementation pose substantial economic and labor burdens. These lengthy and expensive processes are often undervalued or not appealing to the different stake holders who could benefit from validated methods, which lies both within the scientific community (researchers, academic insitutions and funding agencies) as well as outside (*e.g.*, companies) (Vonk et al., 2015). The current regulatory frameworks are being amended in order to allow for more widespread use of NonAMs in safety assessment of chemicals and pharmaceuticals. Yet, the mandatory comparison of NonAMs outcomes with data derived exclusively from animal-based tests remains largely unaddressed (Committee for Medicinal Products for Human Use CHMP, 2023). Consequently, this requirement discourages scientists from progressing beyond the proposal stage of new methods.

### 1.1 European perspective

Committed to reinforcing motivation to implement and validate NonAMs, unifying stakeholders’ efforts, and overseeing the progress, the European Union (EU), via the European Commission (EC), has been at the forefront of promoting the 3R’s for over 30 years (Eur, 2024). The EC’s Horizon 2020 program, succeeded by Horizon Europe, has prompted the establishment of new research avenues which have contingently stimulated the development of NonAMs (Report From The Commission To, 2024). Within EC, the European Research Council (ERC) and the Joint Research Center (JRC) are strongly committed to advancing the 3R’s by providing funding and scientific advice and expertise, respectively. Further intensifying their focus on the 3R’s and NonAMs, the EC has consigned a workforce that will design a focused roadmap for reduce animal testing (Communication From The Commission on, 2023).

The JRC’s EU Reference Laboratory for alternatives to animal testing (EURL ECVAM) is committed to validating and promoting the 3R’s within the scientific community, offering knowledge databases and tools to support and educate scientists across the EU and beyond (Holloway et al., 2021). The European Partnership for Alternative Approaches to Animal Testing (EPAA) aims to improve coordination between the EU and pharmaceutical and cosmetic industries in alignment with the 3R’s (The European Partnership for Alternative Approaches to Animal Testing, 2023). The EU is also developing free e-learning modules through the Education and Training Platform in Laboratory Animal Science (ETPLAS) to promote effective animal research design and education in animal-free methods. Additionally, the European Cooperation in Science and Technology (COST) Action 3R’s Concepts to Improve the Quality of Biomedical Science (IMPROVE, CA21139) focuses on optimizing Structural Funds Programs to enhance research and innovation policies (Ortega Argiles et al., 2023).

Besides these initiatives, there is an increasing amount of 3R’s, centers, associations, hubs, and organizational maps (The European Partnership for Alternative Approaches to Animal Testing, 2023; Johns Hopkins University Center for Alternatives to Animal Testing, 2024; European Animal Research Association, 2022; European Society for Alternatives to Animal Testing, 2023; Norway’s National Consensus Platform for, 2016; European Medicines Agency, 2024; NC3Rs, 2024). Whether the emphasis is on knowledge-sharing through networking and databases or acknowledging the commitment of (young) scientists to the 3R’s, the overarching goal remains clear: bringing researchers together for a future free of laboratory animals.

In the Netherlands in 2016, the *Nationaal Comité advies dierproevenbeleid* (NCad) was tasked to formulate a roadmap for phasing out animal testing procedures (Koëter et al., 2016). One of the recommendations advised the *Ministerie van Landbouw, Natuur en Voedselkwaliteit* (LNV, translated: Dutch Ministry of Agriculture, Nature and Food Quality) to take a guiding role in the process by creating an interdepartmental management group. This led to the inception of the *Transitie Proefdiervrije Innovatie* (TPI) program, focusing on promoting innovation and the gradual acceptance of NonAMs.

### 1.2 Applying the principles of transition science

The transition towards animal-free innovations can be seen through the lens of transition science. The Multilevel Perspective (MLP, Supplementary Figure S1) proposes that change occurs at different levels, such as technology, market, regulations, and societal norms (Geels, 2011). MLP helps to understand that the transition towards animal-free requires new technologies, new ways of working, changes in societal trends, and social pressure. Both building and dismantling activities are necessary for the transition (X-curve principle, Supplementary Figure S1). Many (young) researchers are working on parts of the transition by driving the technological advances, but their are often not aware of its existence, their role in it, nor possess any knowledge on transition theory or what is needed to accelerate it.

According to the transition theory, progressing towards animal-free innovation will typically span over one or two generations (Andreoli et al., 2023). Anticipating that today’s young professionals and students will play a crucial role in facilitating and accelerating the shift towards animal-free science, their early involvement in this process becomes imperative.

In view of these circumstances, Young TPI (also known as Jong TPI and YTPI in some documents) was founded in April 2022 in the Hague and officially became a foundation in October of the same year. Establishing a young network, serving as both supporter and enabler, aligns with recommendations of the NCad and TPI programs.

## 2 The “Why”: mission, vision, and goals

YTPI core values include respect, justice, and courage, which translate into three the lines of action: 1) creating a constructive forum, 2) empowering the network, and 3) exploring (broadening horizons). These align with the fundamental principles of the TPI program. The YTPI network seeks to fortify and expedite this approach by providing open-minded perspectives and raising awareness of animal-free alternatives among young professionals and students of any field whose contributions can support the transition.

“*Contribute to the acceleration of the transition to innovations without laboratory animals by providing open-minded input and raising awareness of animal-free options among young professionals and students”– Mission YTPI*

*“Young TPI empowers the new scientific generation to go animal-(testing)-free”– Vision YTPI*

In the months leading to its official establishment, the “*Young TPI Inception Document*” was defined by the board members, serving as the backbone of the foundation. This comprehensive document, available elsewhere (de Leeuw et al., 2022), outlines the mission and vision statement, target audience, goals, objectives, communication strategy and deliverables. At the moment of its establishment, YTPI is directed by a board of seven young volunteers (Supplementary Figure S2; Supplementary Table S1).

## 3 The “What”: activities

**FIGURE 1:**
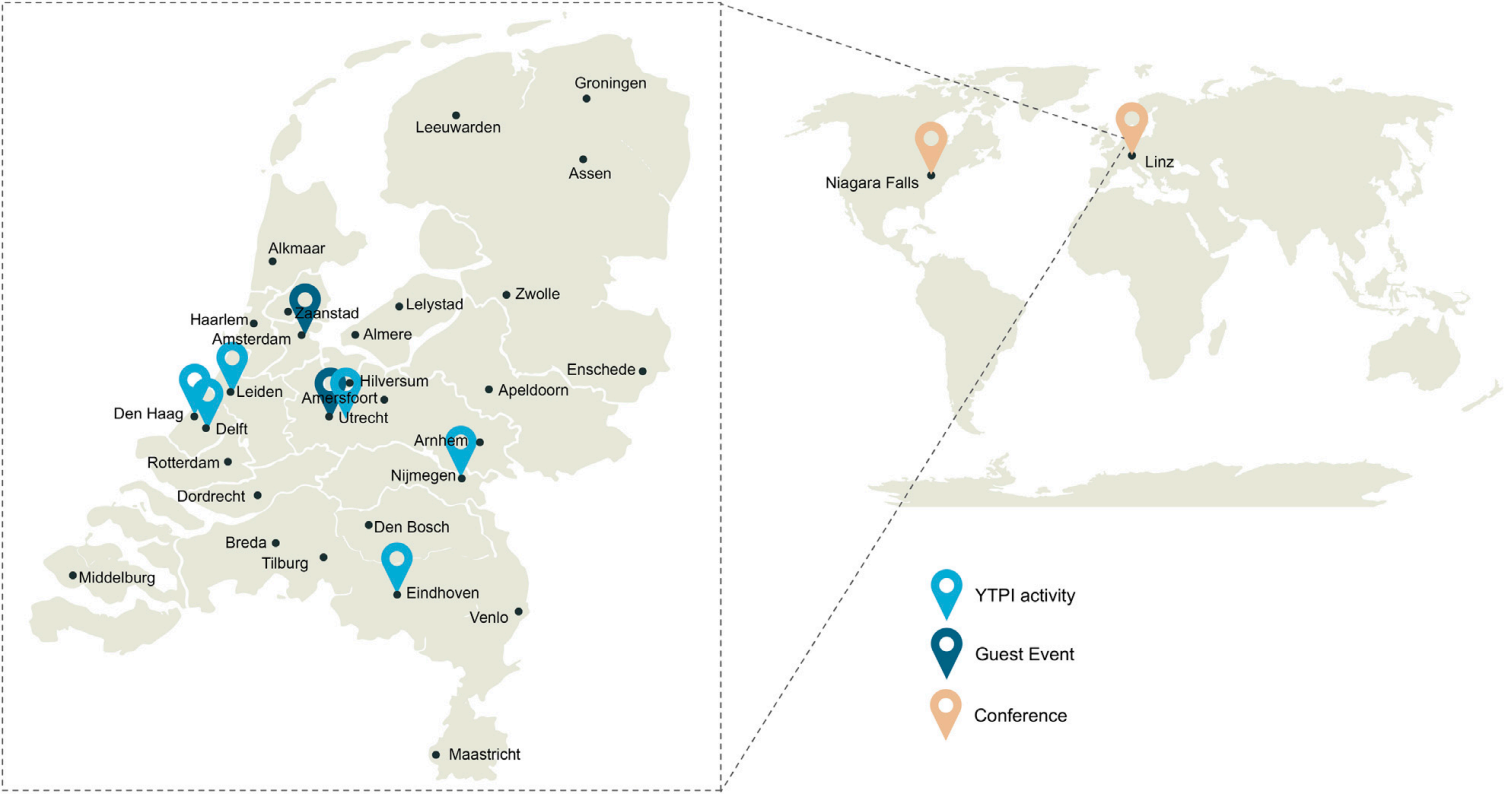
Young TPI activity map. Young TPI focuses mainly on the organization of activities within the Netherlands. More precisely, around big scientific and technological hubs. The first steps towards internationalization have been taken by attending international conferences.

The foundation’s mission of nurturing and expanding the network has found expression through a diverse array of activities and events. These events are a combination of live and online formats including themed activities, guest events, and conferences aligned with the foundation’s objectives. YTPI activities, exclusively organized by the foundation, focused on key goals and were typically tailored for registered members, emphasizing networking opportunities. Additionally, YTPI has been actively participating in partner organized events, contributing with small session. Moreover, (international) conferences have provided strategic platforms to extend the network’s influence and raise awareness on a global scale. In summary, Table 1 lists the events hosted since YTPI’s establishment up to April 2024, while Figure 1 maps their locations, illustrating the breadth of YTPI’s activities.

**TABLE 1:** YTPI’s events and activities from February 2022 to November 2024.

Month	City	Country	Event type	Event title	YTPI goal
2022					
February	Utrecht	NL	Guest lecture	Transition challenge at Veterinary minisymposium	Raise awareness and show the possibilities
March	Utrecht	NL	Guest lecture	Transition challenge BSc Pharmacology	Raise awareness and show the possibilities
July	The Hague	NL	Guest	Organize Youth session at ZonMw Kennisagenda	Raise awareness and show the possibilities
July	Utrecht	NL	Guest lecture	Summer School Advanced In vitro Models	Raise awareness and show the possibilities
September	Linz	AU	Conference	Oral presentation at European Society of Alternatives to Animal Testing	Raise awareness and show the possibilities, Create network and share experiences
September	Utrecht	NL	YTPI event	YTPI launching event: the beginning of the journey	Create network and share experiences
October	Culemborg	NL	Guest lecture	Transition challenge at SGF	Raise awareness and show the possibilities
November	The Hague	NL	Guest	Organize panel session on YTPI at Hugo van Poelgeest	Raise awareness and show the possibilities
December	Leiden	NL	YTPI event	Cozy Brainstorm	Create network and share experiences
2023					
January	Amsterdam	NL	Guest lecture	Transition Challenge at Vrije Universiteit Amsterdam (MSc)	Raise awareness and show the possibilities
February	Online	NL	YTPI event	Webinar: Review or not Review: a powerful tool for NAMs	Stimulate the animal-free transition, Raise awareness and show the possibilities
April	Nijmegen	NL	YTPI event	Night for Animal-free Science	Raise awareness and show the possibilities, Create network and share experiences
May	Utrecht	NL	Guest lecture	Transition Challenge at Hogeschool Utrecht	Raise awareness and show the possibilities
June	The Hague	NL	YTPI event	Talk to TPI	Raise awareness and show the possibilities, Create network and share experiences
September	Delft	NL	YTPI event	YTPI career event	Stimulate the animal-free transition
September	Amsterdam	NL	Guest lecture	Transition Challenge at Vrije Universiteit Amsterdam	Raise awareness and show the possibilities, Create network and share experiences
August	Niagara Falls	CA	Conference	Oral presentation at 12^th^ World Congress on Alternatives and Animal Use in Life Sciences	Raise awareness and show the possibilities, Create network and share experiences
November	Eindhoven	NL	YTPI event	Manifesto Brainstorm	Stimulate the animal-free transition, Raise awareness and show the possibilities, Create network and share experiences
December	Utrecht	NL	Guest lecture	Transition Challenge at Hogeschool Utrecht	Raise awareness and show the possibilities
2024					
February	Online	NL	YTPI event	Webinar: *in vitro* and *in silico* models: match made in heaven?	Stimulate the animal-free transition, Raise awareness and show the possibilities
April	Amsterdam	NL	YTPI event	Presentation of YTPI Manifesto	Stimulate the animal-free transition
May	Utrecht	NL	Guest lecture	Transition challenge at Hogeschool Utrecht	Raise awareness and show the possibilities
May	Utrecht	NL	Guest	Pint of Science Festival	Raise awareness and show the possibilities
June	Online	NL	YTPI event	Webinar: Roadmap to animal free science	Raise awareness and show the possibilities
June	Utrecht	NL	Guest	Young Professionals for the Future of Laboratory Animal and Animal-Free Science	Raise awareness and show the possibilities
September	Leiden	NL	YTPI event	The Future of Food	Raise awareness and show the possibilities; create network and share experiences
September	Linz	AU	Conference	Poster: A manifesto from the young generation advocating to go animal testing-free	Stimulate the transition
October	Utrecht	NL	YTPI event	Workshop: Boosting the Impact of Your Research in Animal-Free Innovation	Raise awareness and show the possibilities; stimulate the transition
October	Amsterdam	NL	Guest lecture	Proefdiervrij lecture	Raise awareness and show the possibilities
November	Amsterdam	NL	Guest lecture	Transition challenge at the Vrij Universiteit Amsterdam	Raise awareness and show the possibilities

## 4 The “Who”: YTPI members

To fulfill its mission, YTPI proposes to foster interactions and collaborations among individuals with diverse educational and professional backgrounds. The network comprises individuals involved in the initial stages of innovation and research with non-animal testing methods, those driving innovations to the market, end-users of innovations, and those responsible for market access and regulatory compliance. Some of the fields represented are: regenerative medicine, stem cell biology, tissue engineering, advanced *in vitro* modeling (*e.g.*, organs-on-chips, organoids), *in silico* studies, omics, artificial intelligence, machine learning, transition science, sustainable development, and environmental science.

BOX 1 **The educational Dutch system defines three routes after secondary education.**

• *Middelbaar beroepsonderwijs* – MBO (middle-level applied education) corresponds to junior college education.

• Higher education comprises of *hoger beroepsonderwijs* – HBO (higher vocational education, provided by *hogescholen*),

• and *wetenschappelijk onderwijs* – WO (scientific/academic education, provided by universities)

**FIGURE 2:**
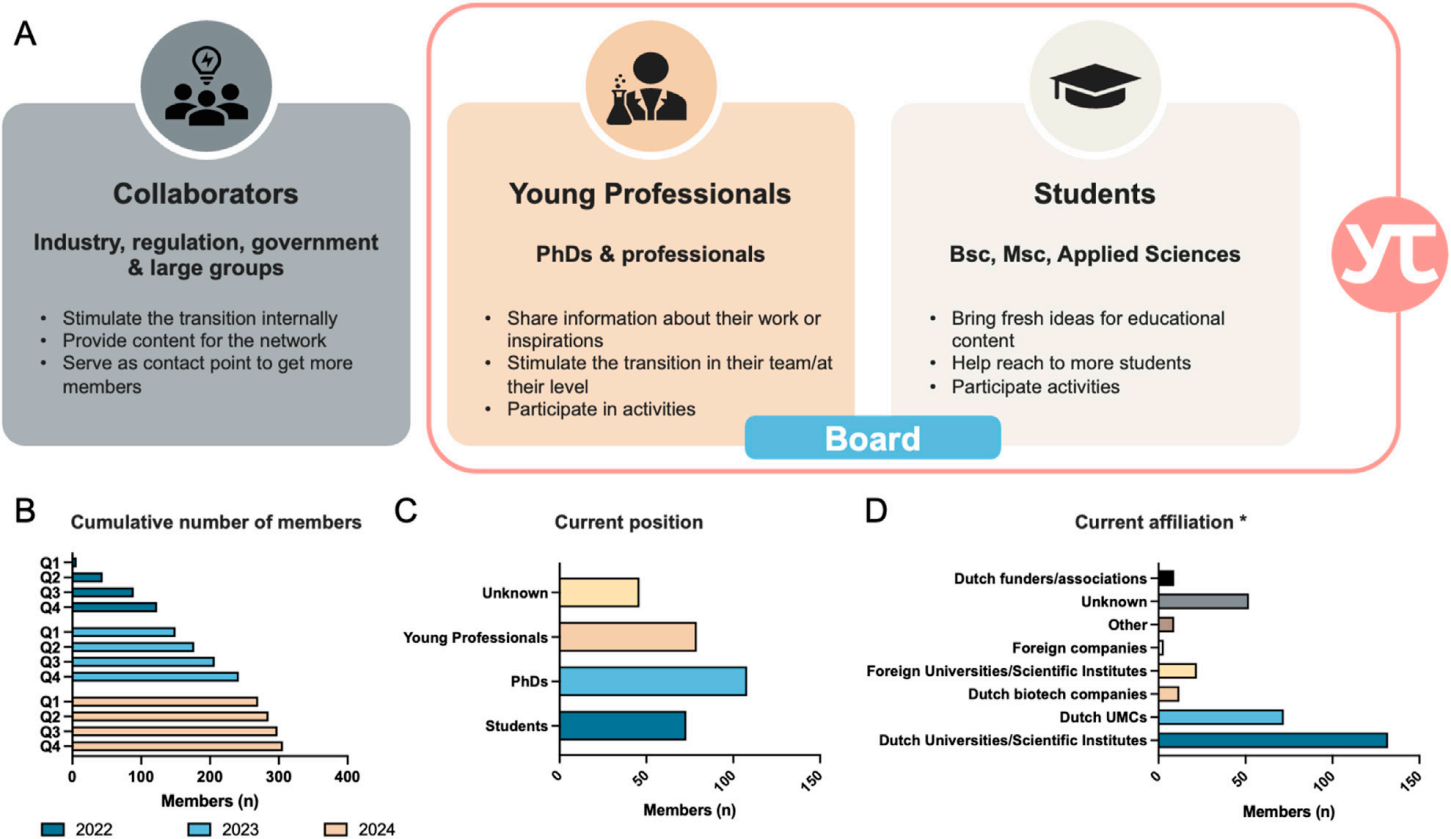
The Young TPI network. **(A)** Young TPI classifies potential members into two distinct groups: collaborators and individual members. While collaborators are in its majority external to the network, they stimulate the transition, provide content for the network and serve as contact points. Moreover, collaborators in the industry have the capability of opening positions within the animal-free research field. The main body of the network is comprised of young professionals and students, who open the discussion and bring new perspectives. They also are the main attendees of the events and sustain and grow the network. **(B–D)** Main findings of the member analysis conducted between February 2022 and November 2024. UMC: University Medical Center. *Some members have double affiliation.

YTPI recognizes two categories of members: collaborators and individual members (Figure 2A). Collaborators encompass organizations throughout the innovation development chain (*e.g.*, drug development, disease modeling, alternative food industry, etc.) contributing with content and opportunities for working in the transition. Individual members are students (MBO, HBO, WO, see Box 1), PhD candidates, and young professionals with a passion for animal-free innovations, animal ethics, medical science, social science, computer science/artificial intelligence, and related fields.

### 4.1 Members

The target audience for membership includes individuals aged 35 years or younger or those who have graduated or defended their master’s degree or PhD not more than 8 years ago. Members can become ambassadors to take an (even more) active role in the network, helping the board with organizational duties. Initially focused on individuals in the Netherlands, YTPI plans to expand its reach to include young professionals from other countries. While the network is open to individuals outside these criteria, they are referred to other structures, such as TPI, local TPI chapters, or equivalents outside the Netherlands, that could better suit their profile.

The growing interest for YTPI is evident in the steadily increasing number of members since the announcement of YTPI establishment in Q1 2022 (Figure 2B). Most members, at the time of registration, are pursuing their PhDs, followed by young professionals and students (Figure 2C). Affiliations are predominantly with Dutch Universities and Medical Centers (Figure 2D). Interestingly, YTPI has also attracted members affiliated outside the Netherlands, underscoring the global demand for young initiatives within the animal-free innovation field. Some members decided not to share their current position and/or affiliation, those appear under *Unknown*.

### 4.2 Ambassadors

The YTPI ambassador program empowers members from various disciplines to promote and represent YTPI within their organizations. This ambassador network fosters engagement and amplifies YTPI’s impact by encouraging participation through peer invitations. Managed by PR, the program involves recruiting new ambassadors, organizing activities, and holding update meetings. Ambassadors support YTPI’s goals by promoting the network, assisting in event planning, creating content, joining think tanks, and representing YTPI. As of April 2024, YTPI has 10 ambassadors from diverse backgrounds across the Netherlands.

### 4.3 Collaborators

YTPI facilitates member collaboration with diverse sector partners -government, society, academia, and business-referred to as “Collaborators.” While collaborators may have diverse perspectives on animal research and biomedical innovation without use of animals, it is precisely these differences that serve as a source of inspiration and *vice versa*. Their inclusion is based on three criteria: alignment with YTPI’s transition goals, active participation in consultation and support, and value-added presence in the network. Additionally, collaborators play a crucial role in linking students and young professionals with the job market within the animal-free innovation field. Key collaborators of YTPI include TPI program, *Stichting Proefdiervrij* and ZonMw.

Besides the collaborators, other organizations, individual scientists, companies, governmental and regulatory bodies that participate in YTPI events are considered “Friends of”. This group includes all speakers and coaches who voluntarily contribute to events due of their personal commitment to the YTPI cause. Among the current “Friends of YTPI”, LUSH (fresh, handmade, animal-free cosmetics brand), Bi/ond (organ-on-chip startup company), MIMETAS (organ-on-chip small company), InnoGI Technologies (formerly The TIM Company, predictive medicine oriented small company), and hDMT (Dutch consortium for organ-on-chip technologies).

## 5 The “When” and “Where”: communication and branding

YTPI leverages social networks to build community, share ideas, and engage members. Primarily using LinkedIn, YTPI shares updates via the TPI network page and hosts a private LinkedIn group for members, ambassadors, and the board to share job posts, events, and discussions. YTPI uses email for formal communications and further event promotion and Instagram (@young_tpi) as an informal channel. The Young TPI website provides event updates and resources. Considering the substantial presence of international members, students and young professionals in the Netherlands, the default communication language is English, with occasional Dutch summaries.

### 5.1 The Young TPI brand

Effective branding is crucial for a non-profit organization like YTPI, establishing a recognizable identity, and attract funding (do Paço et al., 2014). The branding strategy includes a consistent logo, color palette, and badge across all communications and social media, strengthening YTPI’s image (Supplementary Figure S3). These elements are also featured in the “welcoming kit,” consisting of reusable items to decrease the single-use plastics. This comprehensive approach to branding contributes to YTPI’s distinct visual identity, reinforcing its mission and values.

## 6 Future perspectives

YTPI stands out as a ground breaker in the realm of animal-free innovations, being the sole organization exclusively managed by and for young people. Based on the MLP and the transition theory, YTPI strives to raise awareness and showcase the potential of animal-free innovations by educating members on the transition, integrating change management principles, and imparting knowledge on transition science. The efforts to foster collaboration are multi-faceted, encompassing events, trainings, guest lectures, challenges, and international engagement.

At 2 years old, YTPI’s measurable impact remains limited, with current indicators including membership, social media reach, geographic diversity, event attendance, and attendee feedback. YTPI has already contributed to ZonMw’s *Kennisagenda* (translated: knowledge agenda) for the transition to animal-free innovation (Nolte et al., 2023). Once the network’s growth phase has come to an end, and shake-out starts, qualitative and quantitative assessment of the impact in education, policy and the bigger picture of transition will be included.

### 6.1 Education and network growth

The member analysis shows that YTPI’s niche lies in highly educated researchers working at Dutch universities and medical centers in the interface of biomedical research and technology. Expanding the network beyond these groups is key for continuing building on interdisciplinarity and diversity. Regarding the educational level, WO and HBO students in numbers are not as present as desired, despite YTPI’s goal is to attract young researchers. One suggestion is getting YTPI involved in education by, for instance, participating in lectures at all educational levels. Exposure to the transition and the NonAMs at an early stage in the career is fundamental to the paradigm change and has been demanded by YTPI’s members. NonAMs should be introduced in the curricula in a scalable manner, reducing the number of resources (time, expenses) used whilst maximizing the outreach and impact. To this end, mainstream media and social networks present interesting and opportunities worth looking into. Webinars, workshops, conferences and science dissemination activities are already part of the action plan of YTPI and will continue in the action roadmap.

### 6.2 Interdisciplinarity in the transition

Despite that the transition involves several fields of research, disciplines are currently rather isolated. Language barriers, underrepresentation of non-biomedical fields including social sciences, focus on the research “micro-niches,” or lack of financial and strategic support are to blame for the absence of knowledge flow (Young, 2024). YTPI aims to aid in stimulation of interdisciplinary collaborations by ensuring that the not-well-known disciplines (*e.g.*, translational science, regulation, or policy making) are represented in the events. By hosting events which combine scientific research and technology and social sciences topics, it is possible to increase the exposure of both parts.

### 6.3 Policy approach

After the initial push given by governmental bodies, the momentum in policy for animal-free innovation encounters a slowdown. While research is spurring with new cutting-edge technologies and innovations, their implementation is still missing (Abarkan et al., 2022). Funding schemes, incentives, deadlines for the phase-out, laws, and regulatory schemes are falling short in enabling and legitimizing the transition. Yet, the different chemical and drug discovery pipelines will continue to fail until there are better predicting models. Furthermore, the dissemination of information about animal testing alternatives for the non-scientific public is insufficient.

YTPI has the potential to amplify communication by linking young scientists, professionals, and policymakers, broadening mutual understanding of the transition. This can be achieved through lectures, webinars, and conferences.

### 6.4 The bigger picture

The landscape of animal-free innovation is currently technocentric, often neglecting broader considerations such as paradigm shifts, ethical values, and the inherent risks of animal testing. This oversight can obscure the holistic view of the transition away from animal research. Concerns persist about potential human risks, particularly among decision-makers (including senior researchers, journals, sponsors) and citizens, highlighting the need for a comprehensive understanding of the challenges when balancing innovation with ethical responsibility.

The transition to animal-free models introduces various risks. While traditional animal studies assess mechanisms, efficacy, and safety, their replacement raises concerns about increased human risk, especially since the validation of non-animal methods is not yet standardized. Stakeholders may fear reverting to animal models if alternatives fail, after large investments have been done. However, continuing to use animal models carries its own risks due to their limited predictive capabilities. Building confidence in animal-free models will be crucial for this transition.

The ethical justification for animal testing is another pivotal consideration in this transition. It involves assessing the need for additional chemicals and cosmetic products, and pharmaceuticals. The unresolved question is whether animal testing is justified to facilitate the production of more chemicals, new products, cure diseases, and enhance our understanding of biology. This dilemma is further complicated by the risk of stifling innovation, which could potentially impede significant breakthroughs.

Here, Young TPI assumes a crucial role. Going beyond the technological dimension, the network aims to nurture critical thinkers who challenge existing norms and contribute to transformative change. The organization also commits to crafting a persuasive narrative, using its *manifesto* as a foundational document. By amplifying this message through diverse channels and engaging the broader public, Young TPI aims to encourage urgency, highlighting the risks of the *status quo* and illuminating the missed opportunities in the absence of a transition. Young TPI is not merely an organization but a dynamic network for dialogue and collaboration, steering towards a future where innovation thrives without compromising ethical principles.

## 7 Conclusion

Young TPI is a newly created network driven by passionate young people committed to thrust the transition towards animal-free innovations forward. Its events and guest lectures raise awareness across disciplines, influencing career paths and integrating non-animal research into mainstream curricula, challenging traditional animal models. Collaborations with key organizations have enhanced funding opportunities, knowledge exchange, and a supportive ecosystem, connecting like-minded employers and young professionals. YTPI case can only be inspiring for other countries in the evolving landscape of animal-free innovations.

## Data Availability

The datasets presented in this article are not readily available because Member’s personal data is subjected to TPI’s privacy policies (https://www.animalfreeinnovationtpi.nl/privacy). Anonymized statistics compiled for the member analysis is available upon request to the authors.
