# Supplementary material for: Young TPI: empowering animal-free science among the next- generation of scientists
Source: Front Toxicol. 2025 Jan 15;6:1521317. doi: 10.3389/ftox.2024.1521317 (PMC11776087; doi:10.3389/ftox.2024.1521317)

**Young TPI: Empowering animal-free science among the next generation of scientists**

Marta G. Valverde^1,2^, Fatima Zohra Abarkan^1,3^, Rebecca van Eijden^1,4^, Julia M. L. Menon^1,5^, Nikolas Gaio^1,6^, Aarti Ramchandran^1,7^, Victoria C. de Leeuw^1,8^

^1^ Founding Board Member of Young TPI, The Hague, The Netherlands

^2^ Division Pharmacology, Utrecht Institute for Pharmaceutical Sciences, Utrecht, The Netherlands

^3^ Faculty of Science, Radboud University, Radboud Honours Academy, Nijmegen, The Netherlands

^4^ Institute for Management Research, Radboud University, Radboud Honours Academy, Nijmegen, The Netherlands

^5^ Preclinicaltrials.eu, Netherlands Heart Institute, Utrecht, The Netherlands

^6^ BIOND Solutions B.V., Delft, The Netherlands

^7^ MSD Animal Health Netherlands, Boxmeer, The Netherlands

^8^ National Institute for Public Health and the Environment (RIVM), Bilthoven, The Netherlands

Corresponding authors: Marta G. Valverde ([m.garciavalverde@uu.nl](mailto:m.garciavalverde@uu.nl)) and Victoria C. de Leeuw ([victoria.de.leeuw@rivm.nl](mailto:victoria.de.leeuw@rivm.nl) )

**Supplementary Information:**

- **Supplementary Figure 1: Multilevel perspective and S-curve of a transition.**
- **Supplementary Figure 2: Conceptual schematics of YTPI goals and board organigram in 2024.**
- **Supplementary Figure 3: Young TPI branding**
- **Supplementary Table 1: Tasks of the board members and postion holders until 2024**


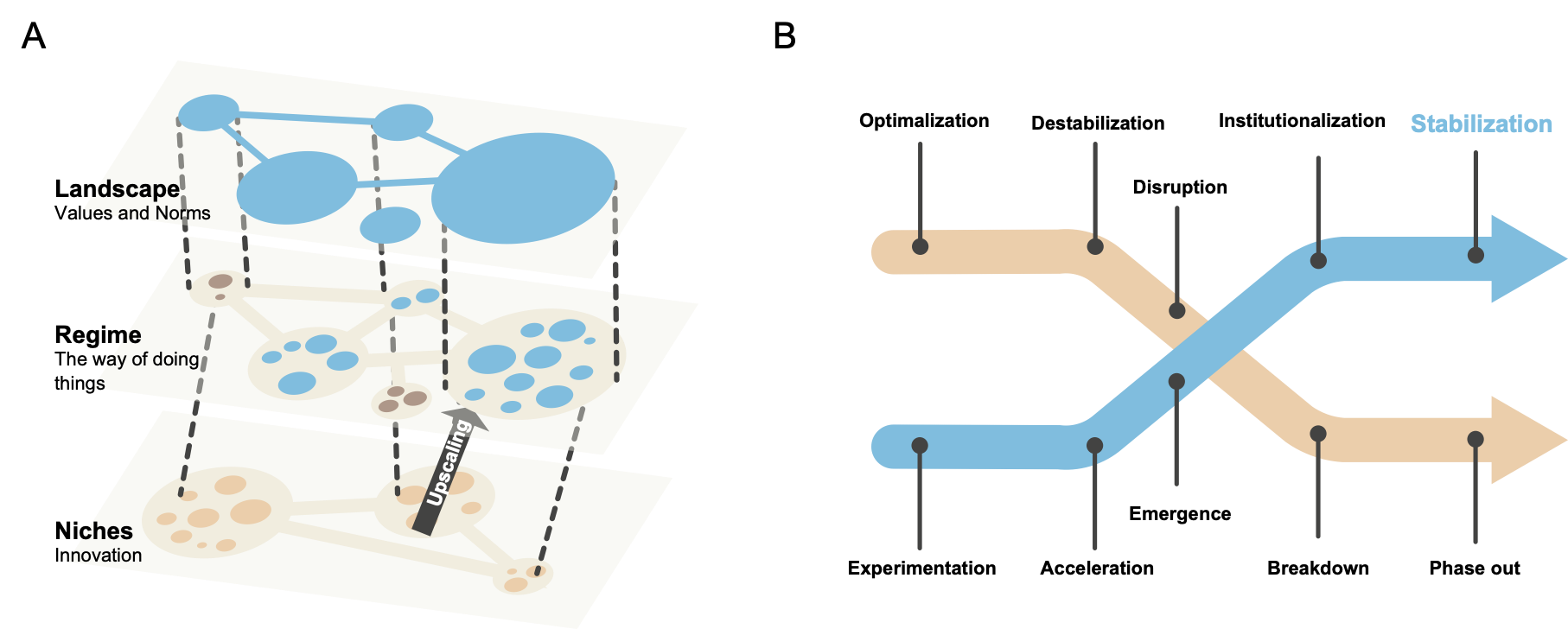
**Supplementary Figure 1: Multilevel perspective and S-curve of a transition.** (A) The multilevel Perspective on transitions is nested, representing that regimes are embedded within landscapes and niches within regimes. Innovation emerges in niches in the context of existing regimes and landscapes with its specific problems, rules, and capabilities. (B) The X-curve model explains the transition dynamics (and its steps) from an old regime (beige) to a new one (pink).


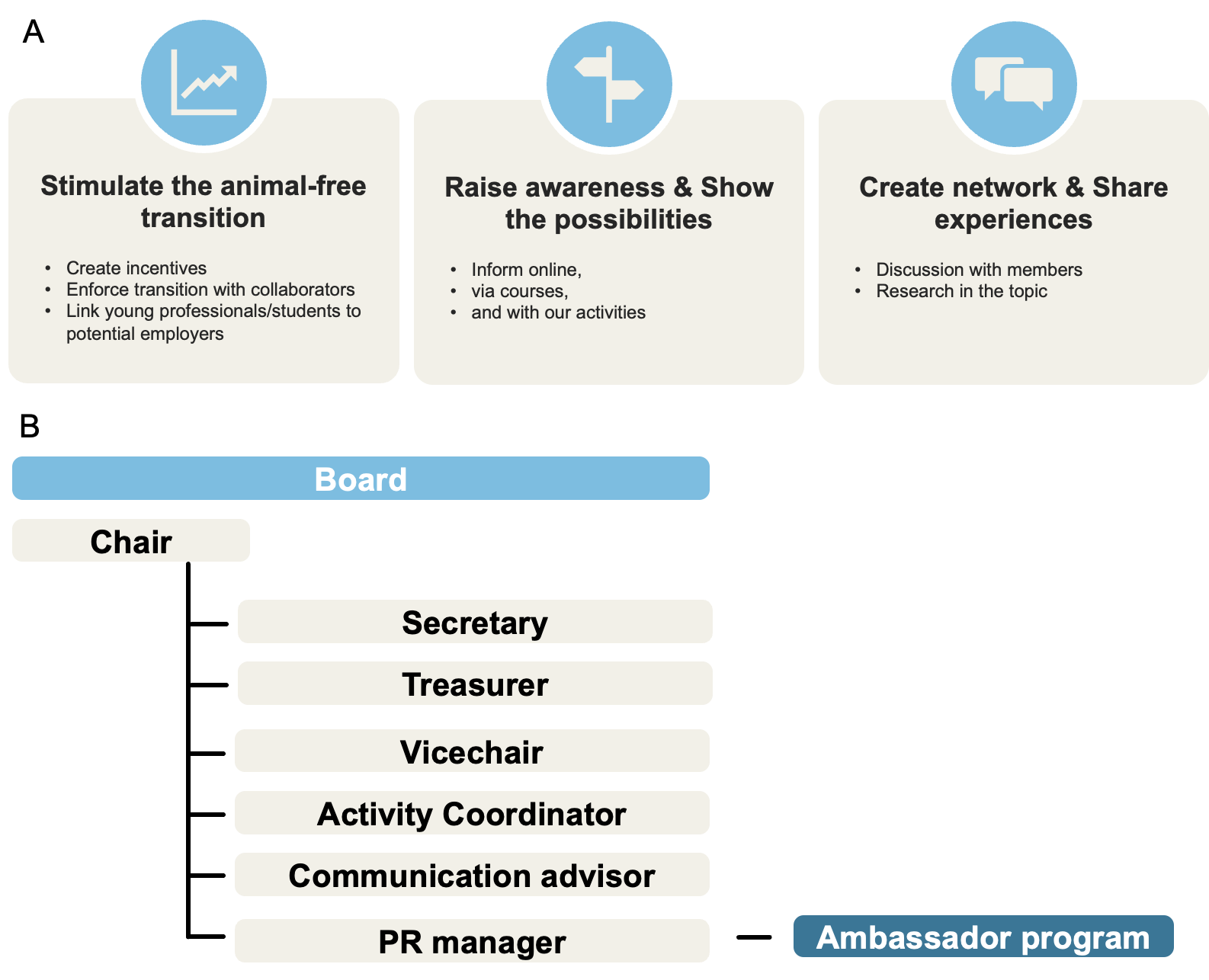


**Supplementary Figure 2:** Conceptual schematics of YTPI goals (A) and board organigram (B) in 2024**.**


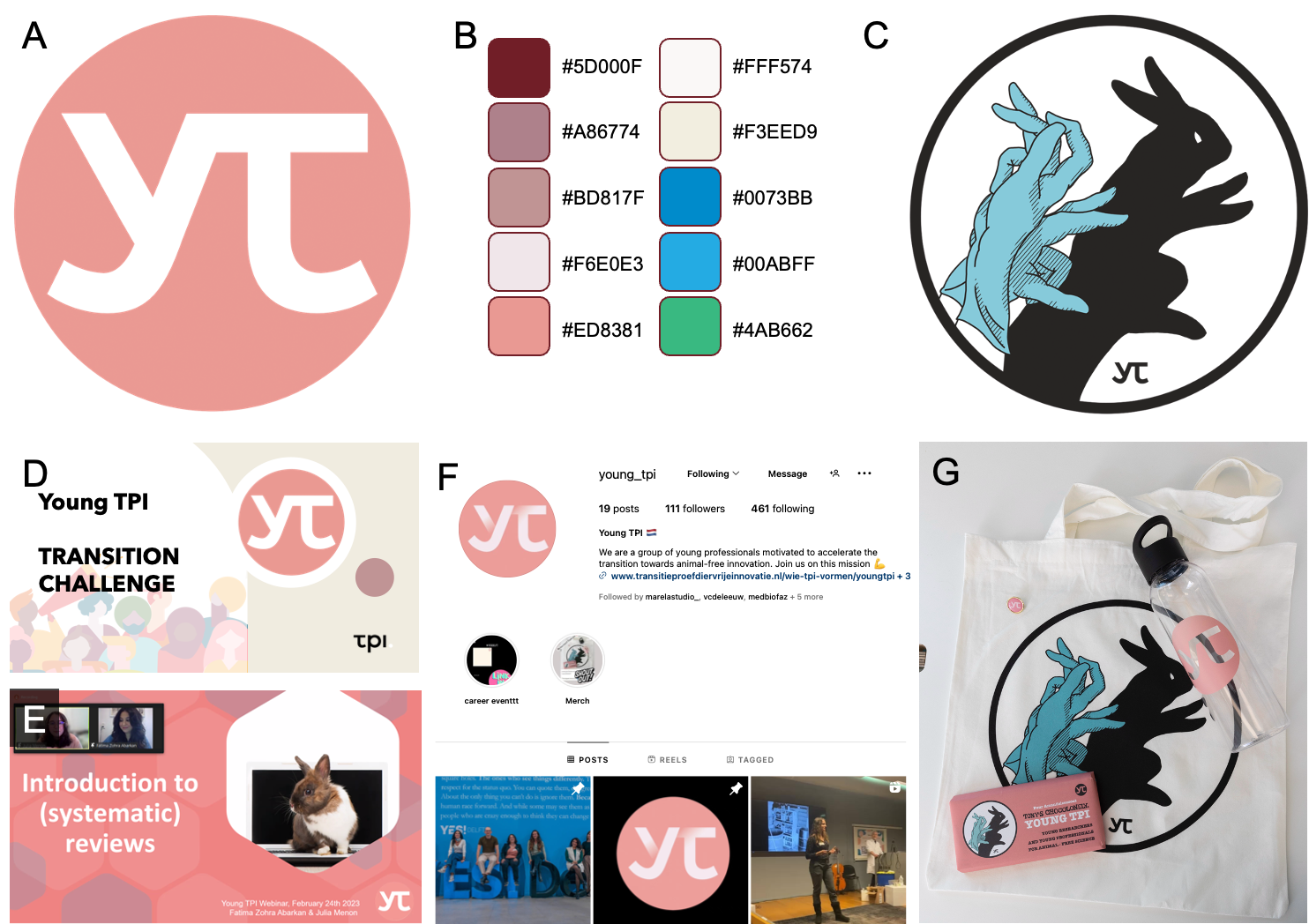


**Supplementary Figure 3: Young TPI branding.** (A) As YTPI aligns with TPI program, the logo takes many of its elements such as the font, the pink color, and the dot. The dot becomes a circle which surrounds a y and a T, standing for Young and TPI, respectively. Smartly, y and T hid the mathematical sign π (PI). (B) With TPI’s pink as signature color, a matching palette is designed. (C) The emblem featuring gloves worn by a scientist and a rabbit was designed with the TPI's "going beyond 3R" concept, trying to put the human (and the scientist) in front. The rabbit is just a shadow and not something concrete, as animal tests are not truly replicating human physiology. The Young TPI brand appears consistently in communications (D,E), merchandise (F) and social networks (G).

**Supplementary Table 1**: Tasks of the board members and postion holders until 2024


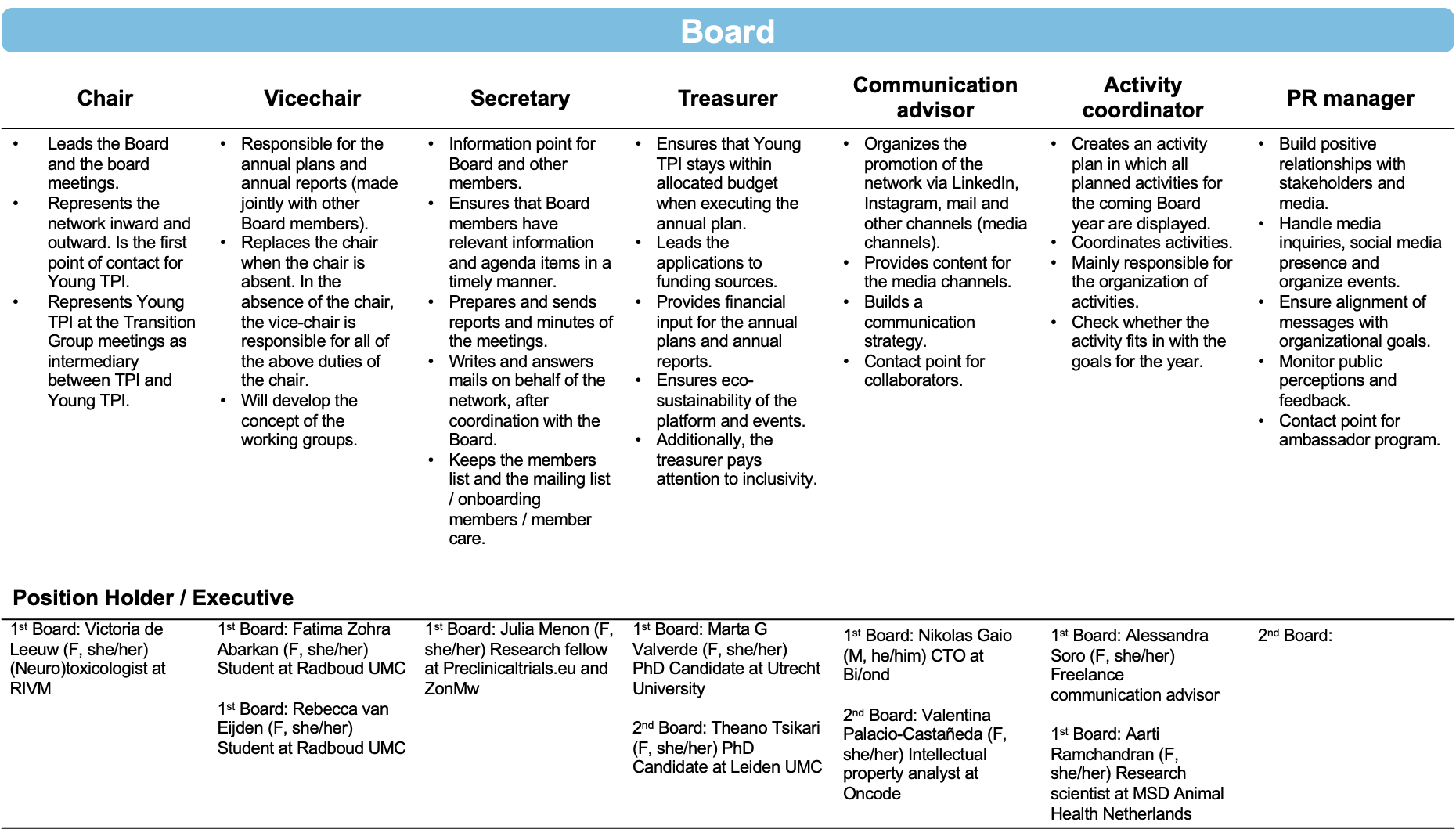

Supplement: Supplementary file 1 [file Table1.docx]
